# The effect of feed and water provision strategies on broiler breeder pullet performance and welfare

**DOI:** 10.3389/fvets.2025.1611967

**Published:** 2025-08-08

**Authors:** Allison D. Weaver, Lisa R. Bielke, Ramon D. Malheiros, Sara K. Orlowski, Allison N. Pullin

**Affiliations:** ^1^Prestage Department of Poultry Science, North Carolina State University, Raleigh, NC, United States; ^2^Department of Poultry Science, University of Arkansas, Fayetteville, AR, United States

**Keywords:** broiler breeder, water use, welfare, drinking behavior, poultry, feed restriction, water restriction, pullet

## Abstract

Feed restriction is common in the broiler breeder industry to optimize health and reproduction. However, this practice has been associated with increased drinking behavior, leading to water spillage, higher litter moisture, and footpad lesions. Consequently, parts of the industry have adopted water restriction protocols. This study aimed to evaluate how different combinations of feed and water restriction affected drinking behavior, welfare, and performance indicators in broiler breeder pullets. At 1 day of age, 960 Cobb 500 FF pullets (*Gallus gallus domesticus*) were randomly allocated to one of four treatments: skip-a-day feeding with *ad libitum* water (SAD + ADLIB), every-day feeding with *ad libitum* water (ED + ADLIB), skip-a-day feeding with 3 h daily water restriction (SAD + WR), and every-day feeding with 3 h daily water restriction (ED + WR). All data were analyzed with generalized linear or linear mixed effects models in R Studio. Drinking behavior was observed at 16 and 22 weeks at an hour after feeding (HAF), when water was turned off for SAD + WR and ED + WR (12:00), and when water access resumed for SAD + WR and ED + WR (14:30). The ED pullets displayed more drinker use at HAF at both ages (*p* = 0.014), while SAD treatments performed more drinker use at 12:00 (*p* < 0.0001) and 14:30 (*p* = 0.0028) at 22 weeks. The WR pullets displayed more drinker use than ADLIB pullets at HAF and 14:30 (*p* < 0.0001), while ADLIB pullets performed more drinker use at 12:00 (*p* = 0.008). Water use (g/bird) was higher in ED + ADLIB pullets at 16 and 22 weeks compared to SAD+ADLIB pullets (*p* = 0.042), but WR groups did not differ (*p* > 0.05). Litter moisture under drinker lines reflected water use patterns, with ED pens wetter at 16 weeks (*p* = 0.0011), but SAD pens unexpectedly had higher moisture at 22 weeks (*p* = 0.011). General pen area litter was wetter in SAD and ADLIB groups (*p* = 0.0036). Footpad scores did not differ among treatments (*p* > 0.05). Body weight and uniformity did not drive water use. Overall, feeding program significantly influenced water use and behavior. Compensatory drinking in WR birds may indicate a welfare concern. Future research should explore measures of satiety and hydration to better understand the behavioral and physiological impacts of water restriction.

## 1 Introduction

Broiler breeders are genetically selected for traits, such as strong appetite and fast growth rate, that result in offspring with high carcass yields (1). However, because broiler breeders have a longer life cycle than their offspring (e.g., 60–70 weeks versus 6–8 weeks), these traits must be carefully managed in breeders to mitigate the risk of developing obesity-related physiological issues, such as tibial dyschondroplasia, ascites, cardiovascular disease, and impaired reproductive function (1–3). Growth management strategies are particularly critical during the pullet phase, where birds are restricted fed in order to follow a recommended growth curve provided by the breeding company to optimize development (4). Feed restriction is implemented throughout the industry to limit nutrient intake either quantitatively or qualitatively (5).

Quantitative feed restriction provides a limited ration with the most common methods being skip-a-day (SAD) or every-day (ED) feeding. A SAD feeding program provides feed on alternate days. This can involve feeding birds every-other-day, on a 4:3 schedule (4 days on feed and 3 days off feed each week), or a 5:2 schedule [5 days on feed and 2 days off feed each week; (4)]. Alternatively, an ED feeding program provides birds with feed at least once daily, but the portions are typically smaller than what would be provided to SAD birds on a feeding day (6). Though feed restriction is necessary to maintain the physiological health status of breeder pullets, the reduction of up to 80% of *ad libitum* feed intake during rearing can result in increased indicators of hunger and poor welfare, such as abnormal repetitive behaviors, stereotypic object pecking, and restlessness (2, 6–8). Providing an ED feeding regimen has been shown to improve both behavioral and physiological metrics through the decrease of feather and object pecking, as well as increased villi height in the small intestine, which is commonly associated with greater nutrient absorption (8–10). However, ED feeding is also associated with more anticipatory activity prior to feeding compared to SAD feeding, suggesting ED-fed birds are less sated due to the smaller portion (11).

Qualitative feed restriction is another feeding method that limits the caloric intake of birds by introducing non-nutritive ingredients to the diet that promote gut fill and satiety (5). For example, insoluble fiber is a feed ingredient that increases the water-holding capacity of digesta within the gut, causing birds to feel sated for longer (12). The addition of fiber in broiler breeder pullet diets has also been noted to decrease feather and object pecking and increase the performance of comfort behaviors, such as preening, dustbathing, scratching, and stretching (13). However, qualitative-restricted diets cannot be fed *ad libitum* as they are not effective at maintaining ideal body weight and uniformity in flocks on their own. Qualitative-restricted diets are often used in combination with quantitative restriction methods (2).

While there is some evidence that qualitative feed restriction reduces the incidence of abnormal behaviors associated with quantitative restriction, indicators of hunger are still apparent (8). For example, excessive drinker use (i.e., polydipsia) is often associated with feed restriction in broiler breeder pullets (6, 14). This may be the result of stereotypic drinker pecking – an abnormal repetitive behavior associated with chronic hunger, where birds redirect pecking from empty feeders onto drinkers and water lines [anecdotally described as “playing” with the drinkers/water lines; (2)]. Prolonged pecking on drinkers can increase the risks for water spillage, higher litter moisture, and higher incidences of pododermatitis (4, 15). However, high drinker use could also be the result of an increased physiological need for water associated with feed restriction practices (16). Nevertheless, water restriction is a method applied to reduce excessive drinker use and subsequently improve litter quality and footpad condition (1). Water lines are often turned off during the pullets’ scotoperiod but may also be turned on and off intermittently throughout the day (16). Though restricting water access may benefit some welfare metrics by maintaining dryer litter, the practice’s effect on animal welfare remains understudied and may exacerbate drinker pecking behavior observed with feed restriction (4). Previous research suggested that limiting water access does not affect physiological metrics of welfare in feed-restricted broiler breeder pullets (17). However, significant genetic developments have been made since this work, and the effect of water restriction on current breeder strains needs further investigation.

The objective of this study was to evaluate the effect of feed and water restriction programs, including SAD versus ED feeding and *ad libitum* versus restricted access to water, on broiler breeder pullet welfare and performance indicators, such as drinking behavior, average bird water use (g/bird), litter moisture, footpad condition, bodyweight, and uniformity. We hypothesized that the most temporally restrictive feed and water provision practices would impair broiler breeder pullet welfare. Specifically, we predicted that SAD-fed and water-restricted pullets would display more drinking behavior and higher water use that may indicate compensatory drinking and/or stereotypic behavior associated with restriction. With higher water use, we also predicted that SAD-fed and water-restricted pullets would have higher litter moisture and consequently a higher prevalence of footpad lesions than ED-fed pullets and pullets with *ad libitum* water access. For performance, we predicted that SAD-fed and water-restricted pullets would maintain lower body weights and a lower coefficient of variation, indicating better uniformity, as these practices are often the preferred method for body weight maintenance in the industry.

## 2 Materials and methods

The North Carolina State University Institutional Animal Care and Use Committee reviewed and approved the experimental procedures for this study (Protocol #23–417). The animals were cared for in accordance with the 4th edition of the Guide for the Care and Use of Agricultural Animals in Research and Teaching.

### 2.1 Animals and housing

A total of 960 female Cobb500 FF (Cobb-Vantress Genetics, Siloam Springs, AR) one-day-old chicks (*Gallus gallus domesticus*) were randomly assigned to one of 16 floor pens (3.8 × 2.4 m, L × W; 60 birds/pen) in a curtain-sided poultry house at the North Carolina State University Chicken Education Unit in Raleigh, North Carolina (60 birds/pen, 1,549.4 cm^2^/bird). For the first 2 weeks of the experiment, mortalities and culls were replaced with extra birds from the same chick flock that were housed in the same location to maintain similar bird numbers across pens.

At placement, birds received 23 h of light, which was decreased by 1–2 h per day until the photoperiod became 8 h (07:00–15:00) by day 14 and remained through 22 weeks of age. Pens were bedded with pine wood shavings (Suncoast Pine Shavings, Fitzgerald, GA). Each pen contained four circular pan feeders (36.8 cm diameter; 2.5 cm/bird total) and one water line with 5 nipple drinkers (12 birds/nipple; Ziggity Systems Inc.^©^, Middlebury, IN). Supplemental feeders and waterers were also provided at chick placement and removed after the first week. At 15 weeks of age, two trough feeders (50.8 cm each) were added to increase feeder space (4.2 cm/bird total). Minimum and maximum house temperatures were recorded every 24 h from one thermometer located on the east side of the house (Supplementary Figure 1). Minimum and maximum external weather temperatures were recorded every 24 h from AccuWeather for Raleigh, NC 27606^1^ (Supplementary Figure 2).

### 2.2 Experimental design

A 2 × 2 factorial design was utilized with two feeding protocol treatments and two water protocol treatments. The feeding treatments consisted of a skip-a-day program where birds were fed on alternate days (SAD) and a program where birds were fed a diet with added fiber once daily (ED). The two water treatments consisted of a protocol with *ad libitum* (ADLIB) water during lights on and a protocol with water restriction intermittently during lights on (WR). The house was divided into quadrants with four pens/quadrant. Due to animal welfare and infrastructure considerations, treatments were unable to be randomly distributed throughout the house. All SAD pens were located on the west half of the house in two quadrants, while all ED pens were located on the east half of the house in two quadrants (8 pens/feeding protocol). The two halves were separated by a 3.6 m storage area, and curtains were hung over the openings to each side of the house. From an animal welfare standpoint, maintaining SAD and ED pens on separate sides of the house reduced visual and auditory stimuli of feeding that could result in anticipation, frustration, and anxiety, particularly for SAD birds during off-feed days. For infrastructure, water clocks were added to water lines to ensure consistency in the timing of when water was turned on and off across treatment pens. However, water clocks were only able to be installed on the water line for each quadrant of the house, so a quadrant of four adjacent pens was assigned to the same water treatment. As a result, one quadrant on each half of the house was assigned to ADLIB and the other was assigned to WR (8 pens/water treatment). The feeding and water protocol treatment combinations included SAD + WR, SAD + ADLIB, ED + WR, and ED + ADLIB (*n* = 4 pens/treatment).

Diets were formulated in consultation with a broiler breeder industry nutritionist to reflect industry practices. All birds were fed the same standard starter diet (Table 1) until 3 weeks of age. At 3 weeks, birds were redistributed to maintain similar bird numbers/pen (58 birds/pen) prior to switching to grower diets and beginning their respective feed and water treatments. Both ED and SAD grower diets had similar nutritional content, but the ED diet had additional fiber to promote gut fill (Table 1). Birds were switched to a developer diet at 16 weeks of age, again with similar overall nutritional content, but increased fiber in the ED diet (Table 1). At the onset of the feeding regimens, SAD birds initially followed a 4:3 feeding program, where they were fed four out of 7 days of the week (Monday, Wednesday, Friday, and Saturday) and had three off-feed days (Tuesday, Thursday, and Sunday). At 14 weeks of age, they were switched to a 5:2 feeding program with off-feed days on Tuesday and Thursday. The ED birds were fed once daily throughout the trial. Feeding for both treatments occurred at approximately 07:30 on respective feed days. Weekly feed quantities were determined for both treatments by following Cobb Genetics (18) recommendations pending age and average bird weight for each pen.

**Table 1:** Composition of pullet diets and calculated analysis.

Ingredients %	Starter	ED	SAD	ED	SAD
		Grower	Grower	Developer	Developer
Ground Corn	58.00	31.20	50.20	54.39	53.44
Soybean Meal 48% CP	25.00	0.55	8.60	2.00	8.75
Wheat Bran	11.12	–	–	–	–
Wheat Midds	–	54.85	30.75	26.04	22.15
DDGS	–	7.20	5.00	11.30	8.00
Limestone Fine	1.63	2.18	2.13	2.16	2.31
MDP	1.77	1.00	1.19	1.20	1.32
Salt (NaCl)	0.25	0.25	0.25	0.25	0.25
Sodium Bicarbonate	0.25	0.25	0.25	0.25	0.25
L-Lysine (50% Liquid)	0.48	0.20	0.07	0.23	0.10
DL-Methionine	0.12	0.20	0.14	0.01	0.07
L-Threonine 98.5%	0.056	–	–	–	–
Mineral Premix^1^	0.20	0.20	0.20	0.20	0.20
Vitamin Premix^2^	0.05	0.05	0.05	0.05	0.05
Arbocel^®^ (cellulose)	–	0.8	–	0.8	–
Selenium premix^3^	0.05	0.05	0.05	0.05	0.05
Choline Chloride	0.02	–	–	–	–
Choline 60% Dry	–	0.02	0.02	0.02	0.02
Phytase	–	–	–	0.05	–
Poultry Fat	1.00	1.00	1.00	1.00	3.00
Total	100	100	100	100	100
Calculated analysis:					
Crude Protein, %	19.00	13.68	14.06	13.40	14.80
Crude Fiber, %	3.30	7.05	4.99	5.64	4.98
Ca, %	1.00	1.18	1.19	1.10	1.18
P, %	0.82	0.86	0.76	0.81	0.80
Available P, %	0.50	0.55	0.50	0.40	0.42
Metabolizable energy, kcal per kg of diet	2850.58	2262.57	2581.89	2623.50	2799.87
Digestible lysine, %	1.34	0.63	0.66	0.63	0.68
Digestible methionine, %	0.42	0.42	0.38	0.23	0.31

^1Mineral premix provides per kg of diet: manganese, 120 mg; zinc, 120 mg; iron, 80 mg; copper, 10 mg; iodine, 2.5 mg; and cobalt.^

^2^Vitamin premix provides per kg of diet: 13,200 IU vitamin A, 4000 IU vitamin D3, 33 IU vitamin E, 0.02 mg vitamin B12, 0.13 mg biotin, 2 mg menadione (K3), 2 mg thiamine, 6.6 mg riboflavin, 11 mg d-pantothenic acid, 4 mg vitamin B6, 55 mg niacin, and 1.1 mg folic acid.

^3^Selenium premix, 1 mg Selenium premix provides 0.2 mg Se (as Na2 SeO3) per kg of diet.

All birds had *ad libitum* access to water until 6 weeks of age. At this time, nighttime water restriction from 15:00 to 07:00 for 16 h total was applied to all pens and continued throughout the remainder of the trial. After a one-week acclimation to nocturnal water restriction, photoperiod water restriction was applied to the SAD + WR and ED + WR pens at 7 weeks of age. Water restricted birds had access to water for the first 4 h of the photoperiod, which included during and after feeding (07:00–11:00), and also for 1 h before the photoperiod ended (14:00–15:00). Water restriction was applied on both on and off-feed days. Water lines were verified to be empty during the daytime restriction period (11:00–14:00). The SAD + ADLIB and ED + ADLIB pens maintained *ad libitum* access to water during the photoperiod. Regardless of water treatment, flow rates were confirmed to be 5.4 ± 2.1 mL/30 s at 4 weeks, 14.6 ± 2.2 mL/30 s at 10 weeks, 12.5 ± 3.4 mL/30 s at 16 weeks, and 12.8 ± 3.1 mL/30 s at 22 weeks across drinker lines.

### 2.3 Drinking behavior

Ten birds were randomly selected at placement to be focal behavior pullets. They were marked with unique colors for identification, using food dye on down feathers at placement and livestock markers after primary feather development (All-Weather Twist-Stik, LA-CO Markal, Elk Grove Village, IL). Markings were touched up as needed to ensure visibility in video recordings. In each pen, one color video camera (4K Smart IP Fixed Bullet Camera, Lorex Corporation, Irvine, CA) was directed at the water line to continuously record video footage for 7 days at 16 and 22 weeks of age. Behavior was observed later in the rearing period as stereotypic behavior tends to increase with age (19). All cameras were linked to a network video recorder (4K 16-channel Wired NVR System, Lorex Corporation, Irvine, CA) to save video files.

At each age, behavior was coded for one on-feed day for all pens using three 20-min segments of video for a total of 60 min/day: an hour after feeding (HAF), time when water was off for WR pens (12:00), and time when water was turned back on for WR pens (14:30). Inter-rater agreement was calculated by comparing drinking durations (sec) between the trainer and trainee for 10 pullets using Microsoft Excel 2016 (version 16.0.5465.1000, Microsoft Corporation, Redmond, WA) to achieve ≥90% inter-rater agreement across all observers.

During video observations, drinking behavior was defined as when the bird’s neck was extended toward the nipple and the beak was in contact with the nipple. The drinking bout ended when beak contact with nipple paused for >3 s, and/or when the bird turned away and its head reached 1 bird length away from the drinker (3.8 cm on the computer screen) or reached the end of the drinker line. Drinking behavior of the 10 focal birds/pen was coded from video files by six trained observers using Behavioral Observation Research Interactive Software [BORIS, version 8.19.3^2^, (20)].

### 2.4 Average bird water use

Water usage for a 48-h period was measured for each pen at 4, 10, 16, and 22 weeks of age using a gravity water system. The two 24-h periods represented an on-feed day and an off-feed day for SAD treatments. At each timepoint, 16.5-gallon carboys were filled, weighed, and placed on top of a ladder in front of each pen (*n* = 4 pens/treatment). Water lines in each pen were disconnected from the barn’s main water supply and reconnected to their respective carboys. For ADLIB pens, carboys were weighed at the end of the photoperiod each day (8 h), prior to manually turning off water flow for nocturnal restriction. For WR pens, carboys were manually turned on and off to maintain the daytime and nocturnal restriction schedule, and they were weighed each time they were turned off. Carboys were refilled and weighed as needed to ensure birds had access to water for the entirety of the treatment’s water access period. Regardless of water treatment, flow rates when connected to carboys were confirmed to be 4.8 ± 1.6 mL/30 s at 4 weeks, 11.5 ± 2.3 mL/30 s at 10 weeks, 9.9 ± 3.6 mL/30 s at 16 weeks, and 10.9 ± 3.6 mL/30 s at 22 weeks across drinker lines.

Pen water use (g/pen) was calculated by subtracting carboy weights from the beginning of the water access period from the end of the access period. The average pullet water use (g/pullet) was determined by dividing the pen water use by the number of birds per pen. The water to feed ratio (W: F) from the on-feed day was also calculated by dividing the average pullet water use by the average feed intake of each pullet (g/pullet).

### 2.5 Litter moisture

At 10, 16, and 22 weeks of age, litter samples were collected from both the general pen area (GEN) and under the water line (WL). For the GEN sample, one handful from each quadrant of the pen was collected 1 m from the water line (4 handfuls/pen) and mixed in a plastic bag for each pen. For the WL sample, one handful of litter was collected directly underneath each of 5 nipples on the water line (5 handfuls/pen) and mixed in a plastic bag for each pen. Approximately 70 g of each sample was weighed then dried in an oven at 100°C for 48-h. Dried samples were reweighed. Litter moisture was calculated using the following equation: (wet weight-dry weight)/wet weight.

### 2.6 Footpad condition scoring

Footpad scoring was performed on the 10 focal birds from each pen at 16 and 22 weeks of age. A scale of 0 to 2 was used (37), with 0 meaning no blemish or discoloration of footpad, 1 indicating small to moderate lesion (<50% of footpad) with black coloring or keratosis of footpad, and 2 meaning severe lesions (≥50% of footpad) and black coloring may extend to toes. Two trained scorers achieved an inter-rater agreement ≥90%.

### 2.7 Body weight and uniformity

Body weights for a subsample of 10 birds per pen were measured weekly from 0 to 22 weeks. Body weights were measured prior to feeding during an on-feed day for all treatments. Birds were weighed individually in a tared container on a ground scale. Weighing did not interfere with data collection of other metrics (i.e., behavior, litter samples, footpad condition), as these metrics were collected on other days in the same week. Coefficient of variation (CV) was calculated from 3 to 22 weeks by dividing the standard deviation of weights by the mean for each pen.

### 2.8 Statistical analysis

All data were analyzed using R statistical software (version 4.4.2; R Core Team, 2021) using RStudio (version 2022.12.0 + 353) for macOS Sonoma 14.7.1. Both generalized linear mixed effects models [glmmTMB package, version 1.1.10, (21)] and linear mixed effects models [nlme package, version 3.1.166, (22)] were used. Generalized linear mixed effects model fits were tested using DHARMa package [version 0.4.7; (23)]. Linear mixed effects model fits were evaluated for normality and homogeneity of variance with visual inspection of quantile-quantile plots and plots of residuals versus fitted values (*qqnorm*, *plot*, and *hist* functions in base R). A stepwise backward reduction using ANOVA for model comparison was used for all analyses to obtain the final models, where *p* > 0.05 was the criterion of exclusion. Linear mixed effects models were fitted using maximum likelihood (ML) to generate a log-likelihood ratio (LLR) test during model comparison, while generalized linear mixed effects models produced a chi-squared (𝜒^2^) test during model comparison. Where there were significant main effects or interactions, differences between specific fixed effects were determined with Tukey’s pairwise comparisons [emmeans package, version 1.10.5, (24)].

Drinking behavior durations were summed by focal bird for each time period (i.e., HAF, 12:00, and 14:30) at 16 and 22 weeks. Separate linear mixed effects models were run for each time period to improve model fit. Feed treatment (factor with two levels: SAD and ED), water treatment (factor with two levels: ADLIB and WR), age (factor with two levels: 16 and 22 weeks), and their 2-way and 3-way interactions were included as fixed effects, and focal bird nested within pen was included as a random effect. Data were square-root transformed to achieve normal distribution of the residual variances.

Pen water usage data were summarized as the average bird water usage/pen/day (g). Separate linear mixed effects models were run for on-feed and off-feed days to improve model fit. Feed treatment (factor with two levels: SAD and ED), water treatment (factor with two levels: ADLIB and WR), age (factor with four levels: 4, 10, 16, and 22 weeks), and their 2-way and 3-way interactions were included as fixed effects. Pen was included as a random effect. Similarly, on-feed W: F data were analyzed using a linear mixed effects model with feed treatment (factor with two levels: SAD and ED), water treatment (factor with two levels: ADLIB and WR), age (factor with four levels: 4, 10, 16, and 22 weeks), and their 2-way and 3-way interactions included as fixed effects. Pen was also included as a random effect.

The proportion of litter moisture was calculated for WL and GEN areas and analyzed using generalized linear mixed effects models with a negative binomial distribution. Separate models were run for WL and GEN areas, where feed treatment (factor with two levels: SAD and ED), water treatment (factor with two levels: ADLIB and WR), age (factor with three levels: 10, 16 and 22 weeks), and their 2-way and 3-way interactions were included as fixed effects. Pen was included as a random effect. Data were analyzed as proportions but are presented as percentages for readability, where model estimates and confidence intervals were multiplied by 100%.

Footpad condition was summarized as the proportion of footpad scores of 0 and 1 in each pen and analyzed using generalized linear mixed effects models with a negative binomial distribution. Separate models were run for scores of 0 and scores of 1. Fixed effects included feed treatment (factor with two levels: SAD and ED), water treatment (factor with two levels: ADLIB and WR), age (factor with two levels: 16 and 22 weeks), and their 2-way and 3-way interactions, with pen as a random effect. Footpad scores of 2 were not statistically analyzed due to their low prevalence (only one bird out of 160 birds scored as 2 at 22 weeks of age). Data were analyzed as proportions but are presented as percentages for readability.

Body weight and CV data were analyzed using linear models to assess the effect of feed treatment and water treatment. Separate models were run for each week with feed treatment (factor with two levels: SAD and ED), water treatment (factor with two levels: ADLIB and WR), and their 2-way interaction included as fixed effects.

## 3 Results

### 3.1 Drinking behavior

**Figure 1:**
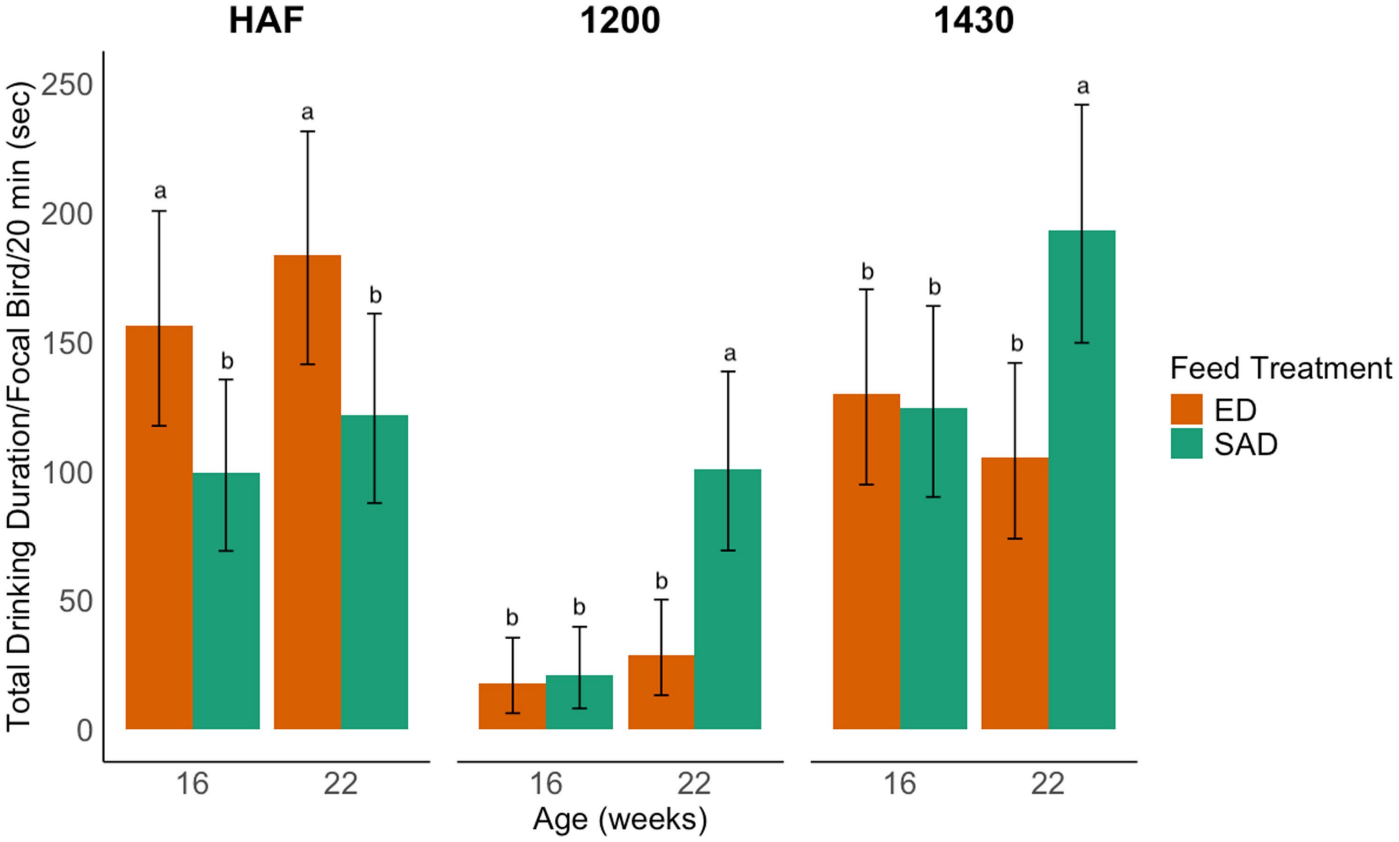
The average drinking duration (sec) by focal broiler breeder pullets at three times of day during an on-feed day. Pullets were fed one of two feeding programs and assessed at 16 and 22 weeks. Bar graphs represent model estimated means ± 95% CI. Letters indicate statistically significant differences identified with a Tukey’s pairwise comparison *post hoc* test (*p* < 0.05). Times of day were sampled at 20-min segments at an hour after feeding (HAF), during the water restriction period for water restricted treatments (12:00), and when the water access resumed for restricted treatments for an hour prior to the photoperiod ending (14:30). Feeding treatments consisted of skip-a-day (SAD) and every-day (ED) feeding programs.

**Figure 2:**
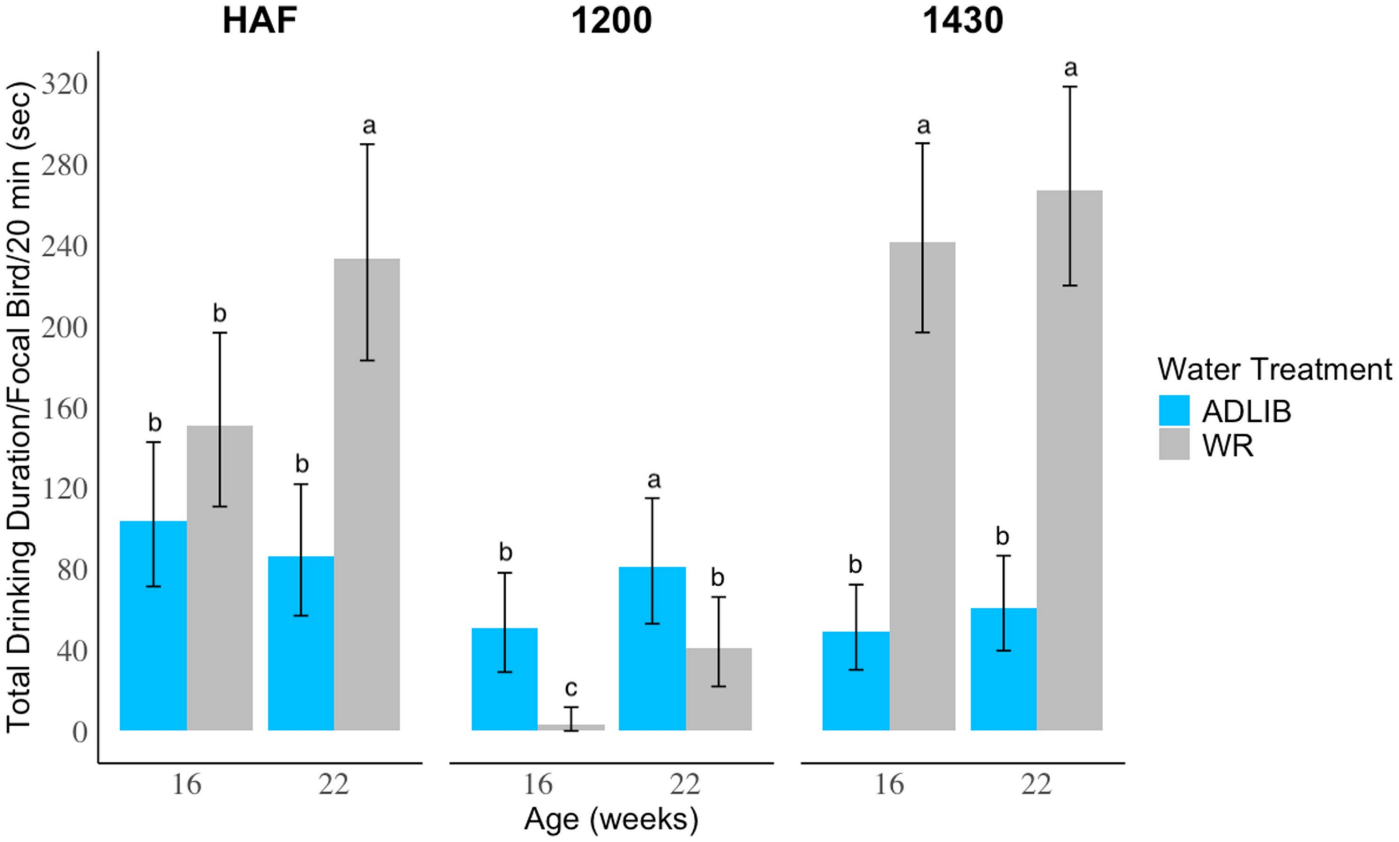
The average drinking duration (sec) by focal broiler breeder pullets at three times of day during an on-feed day. Pullets were reared on one of two water provision programs and assessed at 16 and 22 weeks. Bar graphs represent model estimated means ± 95% CI. Letters indicate statistically significant differences identified with a Tukey’s pairwise comparison *post hoc* test (*p* < 0.05). Times of day were sampled at 20-min segments at an hour after feeding (HAF), during the water restriction period for water restricted treatments (12:00), and when the water access resumed for restricted treatments for an hour prior to the photoperiod ending (14:30). Water treatments consisted of water restriction for 3 h during the 8 h photoperiod (WR) or *ad libitum* (ADLIB) access to water during the 8 h photoperiod.

At HAF, there was a main effect of feed treatment with ED birds spending more time at the drinker than SAD birds at both ages (LLR = 6.10, *p* = 0.0135; Figure 1). There was also a significant interaction between age and water treatment, with WR pullets drinking more than ADLIB pullets at 22 weeks (LLR = 14.61, *p* < 0.0001; Figure 2). There was no feed x water interaction at HAF (LLR = 1.77, *p* = 0.1836). At 12:00, when water was turned off for WR birds, there was an age × feed treatment interaction, with SAD birds spending more time at the drinker than ED birds at 22 weeks (LLR = 16.30, *p* < 0.0001; Figure 1). There was also an age x water treatment interaction at 12:00 where ADLIB spent more time at the drinker than WR birds at both ages, but WR pullets showed a larger increase in drinking time between ages than ADLIB pullets (LLR = 7.03, *p* = 0.008; Figure 2). There was no feed x water interaction at 12:00 (LLR = 0.0073, *p* = 0.93). At 14:30, when water access resumed for 1 h for the WR treatments, there was an interaction between age and feed treatment, with SAD pullets drinking longer than ED pullets at 22 weeks (LLR = 8.94, *p* = 0.0028; Figure 1). A main effect of water treatment was also observed at this time of day, where WR pullets drank more than ADLIB pullets at both ages (LLR = 33.99, *p* < 0.001; Figure 2). There was no feed x water interaction at 14:30 (LLR = 0.60, *p* = 0.44).

### 3.2 Average bird water use

**Figure 3:**
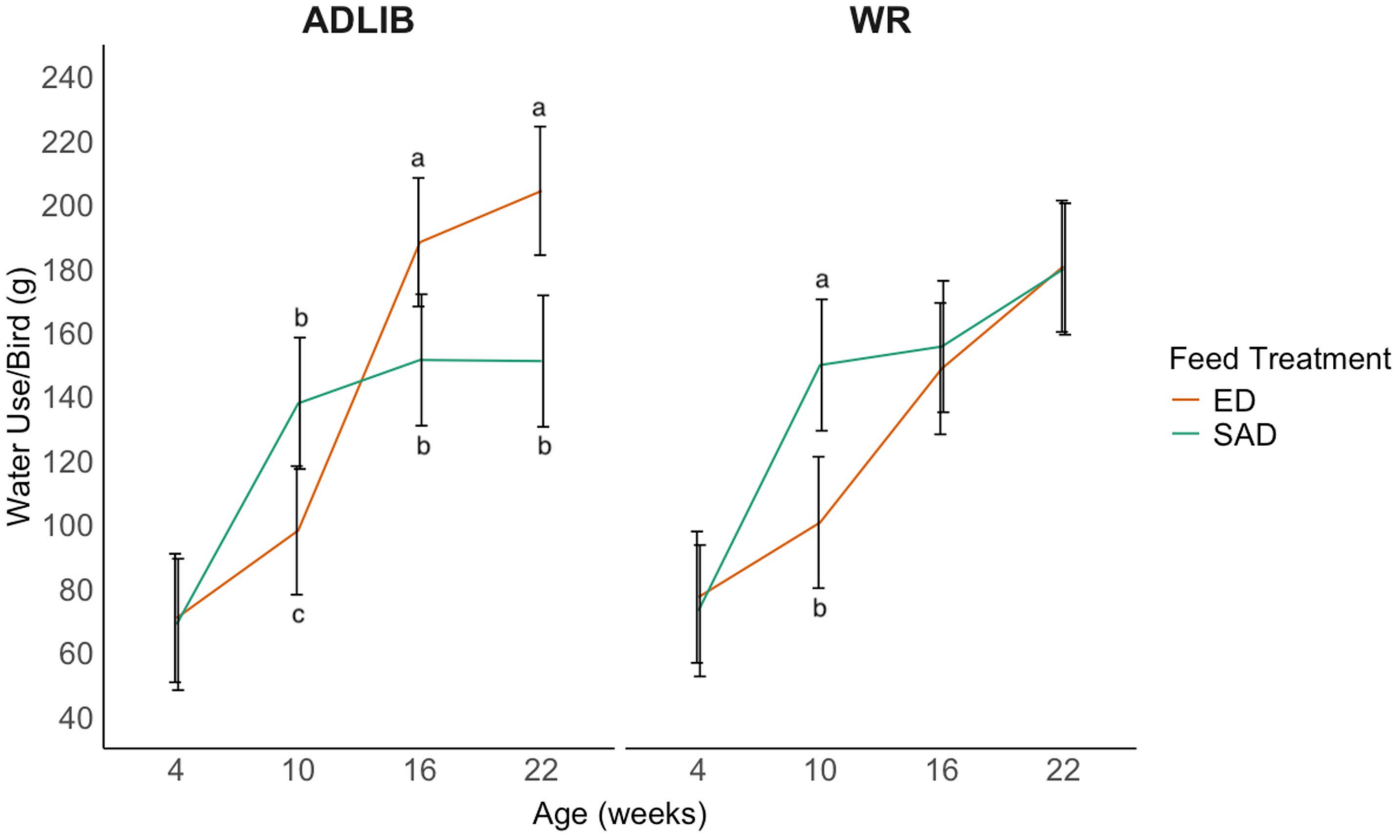
The average water use per bird (g) for broiler breeder pullets during an on-feed day at 4, 10, 16, and 22 weeks. Pullets were reared on one of four feed and water provision treatments in a 2 × 2 factorial design. Line graphs represent model estimated means ± 95% CI. Letters indicate statistically significant differences identified with a Tukey’s pairwise comparison *post hoc* test (*p* < 0.05). Feeding treatments consisted of skip-a-day (SAD) and every-day (ED) feeding programs, while water provision treatments consisted of water restriction for 3 h during the 8 h photoperiod (WR) or *ad libitum* access to water during the 8 h photoperiod (ADLIB).

During the on-feed day, there was a three-way interaction between age, feed treatment, and water treatment (LLR = 8.21, *p* = 0.042; Figure 3). At 4 weeks, water use was similar across all treatments. At 10 weeks, SAD + ADLIB and SAD + WR pullets used more water than ED + ADLIB and ED + WR pullets. With ADLIB water access, ED pullets used more water at 16 and 22 weeks than SAD pullets. However, the WR access resulted in similar water use between SAD and ED pullets at 16 and 22 weeks.

**Figure 4:**
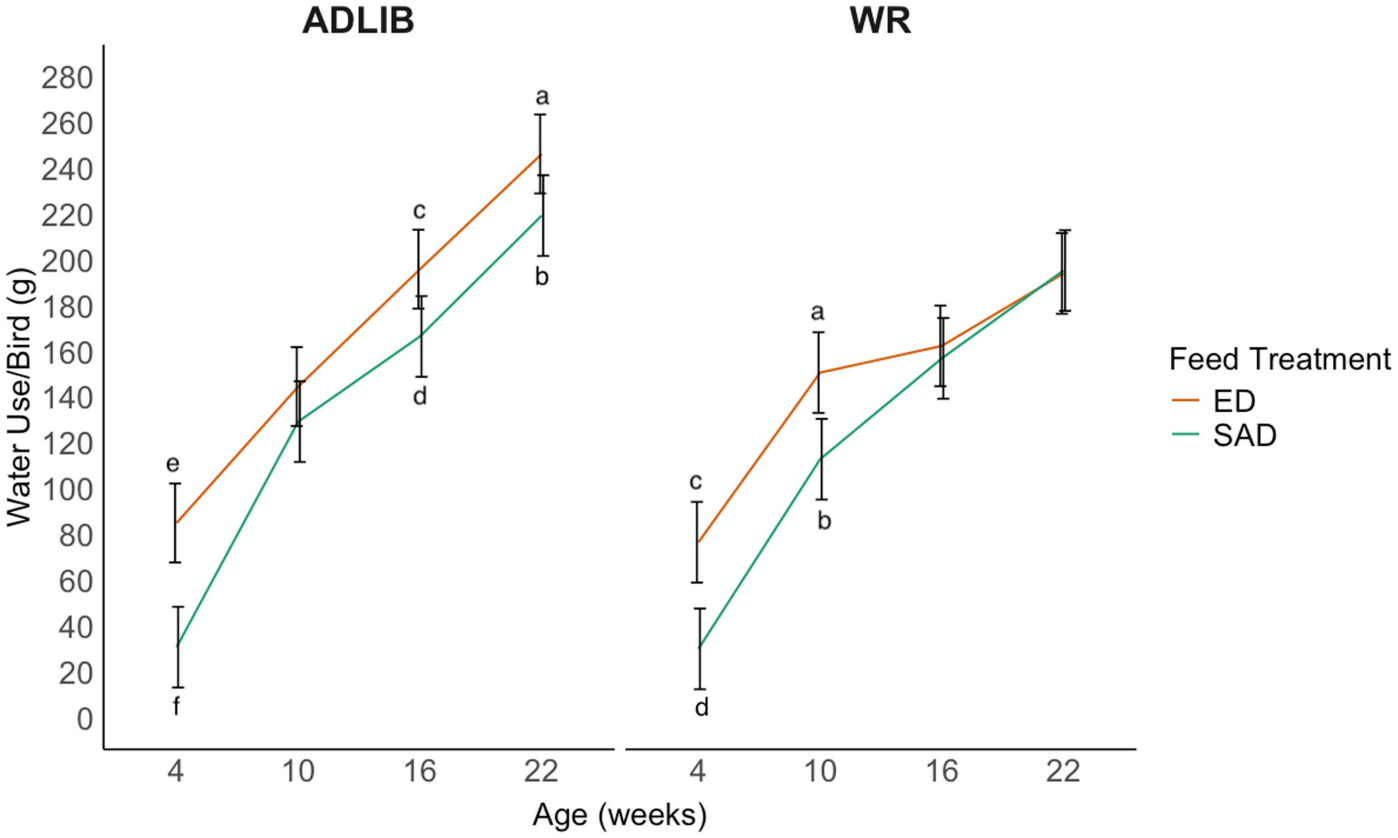
The average water use per bird (g) for broiler breeder pullets during an off-feed day at 4, 10, 16, and 22 weeks. Pullets were reared on one of four feed and water provision treatments in a 2 × 2 factorial design. Line graphs represent model estimated means ± 95% CI. Letters indicate statistically significant differences identified with a Tukey’s pairwise comparison *post hoc* test (*p* < 0.05). Feeding treatments consisted of skip-a-day (SAD) and every-day (ED) feeding programs, while water provision treatments consisted of water restriction for 3 h during the 8 h photoperiod (WR) or *ad libitum* access to water during the 8 h photoperiod (ADLIB).

There was a three-way interaction between age, feed treatment, and water treatment on the off-feed day (LLR = 10.35, *p* = 0.016; Figure 4). At 4 weeks, ED + ADLIB and ED + WR pullets used more water than SAD + ADLIB and SAD + WR pullets. The ED + ADLIB pullets continued using more water than SAD + ADLIB pullets at 16 and 22 weeks, but they used similar amounts of water during week 10. The ED + WR pullets used more water at 10 weeks than SAD + WR pullets; however, there were no differences in water use between the feed treatments at 16 and 22 weeks.

For W: F, there was a two-way interaction between feed treatment and age (LLR = 34.96, *p* < 0.0001; Table 2). During 4, 16, and 22 weeks, ED treatments had a greater W: F than SAD treatments, but W: F was similar at 10 weeks. There were no other significant interactions or effects.

**Table 2:** Water to feed ratio estimate (95% CI) calculated from average bird water intake (g) and average bird feed intake (g) of broiler breeder pullets reared on one of four feed and water provision treatments in a 2 × 2 factorial design^1^.

Treatment	4 weeks	10 weeks	16 weeks	22 weeks
Feed				
ED	2.00 (1.83, 2.17)^a^	1.70 (1.52, 1.87)	1.89 (1.72, 2.06)^a^	1.59 (1.42, 1.76)^a^
SAD	1.16 (0.99, 1.33) ^b^	1.47 (1.30, 1.64)	1.31 (1.13, 1.48)^b^	0.98 (0.81, 1.15)^b^
Water				
ADLIB	1.52 (1.35, 1.69)	1.54 (1.37, 1.71)	1.70 (1.53, 1.87)	1.29 (1.12, 1.46)
WR	1.64 (1.47, 1.81)	1.63 (1.46, 1.80)	1.50 (1.33, 1.67)	1.28 (1.11, 1.45)

^1^Feeding treatments consisted of skip-a-day (SAD) and every-day (ED) feeding programs, while water provision treatments consisted of water restriction for 3 h during the 8 h photoperiod (WR) or *ad libitum* (ADLIB) access to water during the 8 h photoperiod.

Superscripts differing within a column indicate statistically significant differences identified with a Tukey’s pairwise comparison *post hoc* test (*p* < 0.05).

### 3.3 Litter moisture

There was a three-way interaction between feed treatment, water treatment, and age for the WL samples (𝜒^2^ = 13.67, df = 2, *p* = 0.0011; Table 3). At 10 weeks, ED + ADLIB pens had the highest WL litter moisture, followed by the other three treatments with a similar moisture level. At 16 weeks, ED + ADLIB had the highest WL litter moisture again, with SAD + ADLIB and ED + WR in the middle and SAD + WR with the lowest litter moisture. However, by 22 weeks the SAD + ADLIB pens had the highest litter moisture, followed by the ED + ADLIB and SAD + WR, with the ED + WR having the lowest WL litter moisture.

**Table 3:** Litter moisture percent estimate (95% CI) sampled directly underneath the water line in pens of broiler breeder pullets reared on one of four feed and water provision treatments in a 2 × 2 factorial design^1^.

Treatment	10 weeks	16 weeks	22 weeks
ADLIB			
ED	35.5 (30.8, 40.6)^a^	52.6 (47.3, 57.8)^a^	28.3 (24.1, 32.9)^b^
SAD	21.7 (18.1, 25.7)^b^	34.6 (29.9, 39.5)^b^	35.2 (30.5, 40.2) ^a^
WR			
ED	17.8 (14.6, 21.4)^b^	37.9 (33.1, 43.0)^b^	22.0 (18.4, 26.1)^c^
SAD	19.7 (16.3, 23.6)^b^	25.2 (21.3, 29.6)^c^	29.2 (24.9, 33.8)^b^

^1^Feeding treatments consisted of skip-a-day (SAD) and every-day (ED) feeding programs, while water provision treatments consisted of water restriction for 3 h during the 8 h photoperiod (WR) or *ad libitum* (ADLIB) access to water during the 8 h photoperiod.

Superscripts differing within a column indicate statistically significant differences identified with a Tukey’s pairwise comparison *post hoc* test (*p* < 0.05).

For GEN litter samples, there was an age x feed interaction, where SAD-fed birds had increased litter moisture compared to ED birds at 10 weeks only, and litter moisture content was similar between these treatments at 16 and 22 weeks (𝜒^2^ = 11.25, df = 2, *p* = 0.0036; Table 4). There was also a main effect of water, with ADLIB pens having greater GEN moisture compared to WR pens at all ages (𝜒^2^ = 7.88, df = 1, *p* = 0.005; ADLIB: 22.90 [21.10, 24.80], WR: 18.90 [17.30, 20.60] %, mean [95% CI]).

**Table 4:** General litter moisture percent estimate (95% CI) sampled 1 m away from the water line in pens of broiler breeder pullets reared on a skip-a-day (SAD) or every-day (ED) feeding program.

Treatment	10 weeks	16 weeks	22 weeks
ED	14.7 (12.8, 16.9)^b^	28.0 (25.4, 30.9)	19.5 (17.3, 22.0)
SAD	18.7 (16.5, 21.1)^a^	25.1 (22.6, 27.8)	21.0 (18.7, 23.5)

Superscripts differing within a column indicate statistically significant differences identified with a Tukey’s pairwise comparison *post hoc* test (*p* < 0.05).

### 3.4 Footpad condition scoring

There was no effect of feed treatment, water treatment, or age on footpad condition scores of 0 (*p* > 0.05; Table 5). Similarly, for footpad condition scores of 1, there were also no significant interactions or main effects of feed treatment, water treatment, or age (*p* > 0.05; Table 5).

**Table 5:** The percentage (95% CI) of broiler breeder pullets in footpad score categories^1^.

Footpad score	Treatment	16 weeks	22 weeks
0	ED + ADLIB	95.3 (86.3, 98.5)	93.2 (83.6, 97.3)
	SAD + ADLIB	95.1 (85.6, 98.5)	92.9 (81.6, 97.5)
	ED + WR	91.6 (80.0, 96.8)	88.0 (75.1, 94.7)
	SAD + WR	91.3 (79.1, 96.7)	87.6 (72.2, 95.0)
1	ED + ADLIB	5.5 (1.8, 15.8)	6.9 (2.5, 17.3)
	SAD + ADLIB	4.8 (1.6, 14.0)	6.0 (2.1, 16.3)
	ED + WR	8.8 (3.3, 21.5)	10.8 (4.5, 23.9)
	SAD + WR	7.7 (2.9, 19.2)	9.5 (3.6, 22.6)

^1^Footpad score of 0 denotes no blemish or discoloration of the footpad. Footpad score of 1 indicates small to moderate lesion (<50% of footpad) with black coloring or keratosis of footpad.

Pullets were reared on one of four feed and water provision treatments in a 2 × 2 factorial design. Feeding treatments consisted of skip-a-day (SAD) and every-day (ED) feeding programs, while water provision treatments consisted of water restriction for 3 h during the 8 h photoperiod (WR) or *ad libitum* (ADLIB) access to water during the 8 h photoperiod.

No statistical differences were identified (*p* > 0.05).

### 3.5 Body weight and uniformity

For body weights, both ED treatments were significantly higher than both SAD treatments regardless of water provision at 8, 12, 13, 17, 19, and 20 weeks (Table 6). In contrast, both SAD treatments had higher body weights than both ED treatments at 22 weeks (Table 6). There was an interaction between feed and water treatments at 18 weeks, with the ED + ADLIB treatment having the highest body weights, followed by ED + WR, SAD + WR, and SAD + ADLIB (Table 6). There was another interaction between feed and water at 21 weeks as SAD + ADLIB had greater body weights than ED + ADLIB (Table 7). There were no significant effects or interactions at any of the other ages.

**Table 6:** Individual bird body weight (kg) estimates for broiler breeder pullets reared on one of four feed and water provision treatments in a 2 × 2 factorial design^1^.

Week	Treatment				SEM^2^	*P*-values		
	ED + ADLIB	ED + WR	SAD + ADLIB	SAD + WR		Feed	Water	Feed × Water
3	0.56	0.54	0.55	0.55	0.019	0.84	0.56	0.83
4	0.59	0.57	0.58	0.58	0.017	0.84	0.63	0.83
5	0.64	0.62	0.63	0.62	0.019	0.84	0.57	0.89
6	0.69	0.68	0.69	0.68	0.021	0.94	0.80	0.87
7	0.75	0.76	0.76	0.76	0.021	0.78	0.80	0.78
8	0.85^a^	0.89^a^	0.80^b^	0.85^b^	0.016	0.046*	0.089	0.99
9	0.94	0.95	0.92	0.93	0.024	0.66	0.64	0.82
10	1.02	1.04	1.02	1.01	0.027	0.97	0.60	0.62
11	1.11	1.10	1.09	1.07	0.025	0.63	0.92	0.71
12	1.23^a^	1.23^a^	1.15^b^	1.20^b^	0.019	0.012*	0.88	0.18
13	1.33^a^	1.34^a^	1.23^b^	1.32^b^	0.021	0.0073*	0.59	0.14
14	1.43	1.41	1.34	1.41	0.027	0.053	0.69	0.15
15	1.54	1.60	1.53	1.51	0.037	0.86	0.24	0.26
16	1.69	1.75	1.67	1.64	0.038	0.72	0.23	0.24
17	1.89^a^	1.86^a^	1.67^b^	1.71^b^	0.024	<0.0001*	0.42	0.14
18	2.08^a^	1.95^b^	1.79^d^	1.87^c^	0.024	<0.0001*	0.0023*	0.0010*
19	2.18^a^	2.16^a^	1.95^b^	2.08^b^	0.037	0.0008*	0.69	0.061
20	2.30^a^	2.35^a^	2.09^b^	2.19^b^	0.050	0.011*	0.52	0.60
21	2.41^b^	2.54^ab^	2.63^a^	2.57^ab^	0.043	0.0035*	0.052	0.044*
22	2.58^b^	2.71^b^	2.72^a^	2.75^a^	0.042	0.040*	0.053	0.27

^1^Feeding treatments consisted of skip-a-day (SAD) and every-day (ED) feeding programs, while water provision treatments consisted of water restriction for 3 h during the 8 h photoperiod (WR) or *ad libitum* (ADLIB) access to water during the 8 h photoperiod.

^2SEM, standard error of the mean.^

Superscripts differing within a row indicate statistically significant differences (*p* < 0.05).

* indicates *p* < 0.05.

**Table 7:** Average coefficient of variation (%) estimates for pens of broiler breeder pullets reared on one of four feed and water provision treatments in a 2 × 2 factorial design (*n* = 4 pens/treatment)^1^.

Week	Treatment				SEM^2^	*P*-values		
	ED + ADLIB	ED + WR	SAD + ADLIB	SAD + WR		Feed	Water	Feed × Water
3	9.32	11.57	10.08	10.24	1.85	0.78	0.41	0.58
4	9.32	10.49	10.34	8.81	1.70	0.68	0.63	0.44
5	10.23	10.98	10.78	9.88	1.19	0.76	0.67	0.51
6	11.36	10.74	11.28	10.12	1.51	0.97	0.78	0.86
7	13.04	11.09	11.59	7.84	0.85	0.25	0.13	0.31
8	14.62	11.06	11.33	10.6	1.23	0.08	0.06	0.26
9	14.06^a^	10.96^b^	12.32^a^	8.55^b^	0.88	0.19	0.029*	0.71
10	13.87	11.30	12.85	9.49	1.18	0.55	0.15	0.74
11	15.50	12.60	16.27	13.59	2.84	0.85	0.49	0.97
12	13.38	12.36	15.09	12.64	1.68	0.49	0.67	0.68
13	10.05	12.15	13.62	12.19	1.24	0.07	0.26	0.18
14	16.60	13.12	15.79	11.03	1.38	0.68	0.10	0.65
15	11.49	12.68	12.16	11.71	1.23	0.71	0.51	0.52
16	11.96	12.78	12.08	11.14	1.33	0.95	0.67	0.52
17	11.74	15.48	16.03	13.59	2.43	0.24	0.30	0.23
18	16.12	16.64	15.77	16.82	2.33	0.92	0.88	0.91
19	10.05^b^	13.13^bc^	14.50^a^	9.72^c^	1.36	0.040*	0.14	0.014*
20	10.42	11.27	12.81	10.01	2.06	0.43	0.77	0.39
21	10.87	12.09	10.70	10.38	1.34	0.93	0.53	0.57
22	11.44	11.73	10.15	11.89	1.75	0.61	0.91	0.69

^1^Feeding treatments consisted of skip-a-day (SAD) and every-day (ED) feeding programs, while water provision treatments consisted of water restriction for 3 h during the 8 h photoperiod (WR) or *ad libitum* (ADLIB) access to water during the 8 h photoperiod.

^2SEM, standard error of the mean.^

Superscripts differing within a row indicate statistically significant differences (*p* < 0.05).

* indicates *p* < 0.05.

For uniformity there was a main effect of water treatment at 9 weeks, with both ADLIB treatments having a higher CV than both WR treatments (*p* = 0.029; Table 7). There was an interaction between feed and water treatments at 19 weeks, where SAD + ALIB had a higher CV than SAD + WR and ED + ADLIB (*p* = 0.014; Table 7). There were no treatment effects at any other age (*p* > 0.05; Table 7).

## 4 Discussion

### 4.1 Drinking behavior

We anticipated that SAD and ED-fed pullets would show differences in drinking behavior at different timepoints throughout the on-feed day due to differences in quantity of feed and fasting periods, which was observed. The ED-fed birds drank more at HAF at both ages, while SAD-fed birds drank more at 12:00 and 14:30 at 22 weeks. This is most likely because ED-fed pullets have a smaller quantity of feed to consume than SAD-fed pullets, so they finished eating and began drinking earlier. In contrast, SAD-fed birds have a larger quantity of feed to consume on feed days, particularly as they age. Larger feed quantities lead to more overall water use, which has been noted in SAD-fed birds when compared to ED-fed birds in previous research (16). However, it is important to consider the timing of this increased drinking behavior when examining the implications of water restriction practices used alongside SAD or ED feeding programs. Because of the larger feed rations, SAD-fed birds might not be finished consuming their feed at HAF, thus drinking more at later times in the day. Our findings suggest that, for SAD-fed pullets, restricting access to water even several hours after feeding could prevent them from fulfilling physiological needs and risking adequate hydration (25).

We also expected differences in drinking behavior between ADLIB and WR treatments, with a prediction that WR pullets would display more drinking behavior than ADLIB pullets due to compensatory drinking when they had access to water. These results were observed, as WR birds exhibited more drinking behavior at 14:30 at both ages while ADLIB birds displayed more drinking behavior more at 12:00 at both ages. Water restriction practices are often implemented simultaneously with feed restriction to prevent water spillage from birds “playing” with the drinkers (15). We hypothesized that “playing” with drinkers was stereotypic pecking behavior directed toward the drinker line, which could be indicated by birds continuing to peck at the drinker even when the water line is not on (6). The results from our study indicate that the WR protocols were successful in minimizing drinking behavior when water was turned off, as pullets learned the WR schedule and mostly did not engage with the drinker when the water was off. Water output (i.e., water being expelled from the nipple) appears to be a critical component of satisfying the pecking behavior at the drinker line, which indicates a function of the behavior to obtain water. Stereotypic behaviors are defined as “repetitive and unvarying while not serving an obvious function” (26). In the current study, pecking at the drinker line to display drinking behavior appears to serve a function of obtaining water and may not be stereotypic. However, despite 12 weeks of training on the WR schedule, the WR pullets increased their drinking behavior attempts at 12:00 at 22 weeks, possibly reflecting a greater need for water at this age or the development of stereotypic behavior as the birds aged.

While the WR protocol was effective in minimizing drinking behavior when water was off, the WR pullets displayed an increase in drinking behavior at 14:30, indicating compensatory drinking when the water was turned back on. WR treatments also displayed more drinking behavior than ADLIB treatments at HAF at week 22, potentially indicating an anticipatory reaction to losing water access in combination with increased ambient temperatures. Increased water consumption after a period of deprivation has been previously observed in both broilers and broiler breeders (16, 25, 27). Bennett and Leeson (16) noted that SAD birds with *ad libitum* access to water actually drank less on an off-feed day than SAD-fed birds with WR, similarly suggesting that water restriction elicits compensatory consumption. Also, broiler breeder pullets with 3 h of water access used twice as much water than pullets with 5 h and 8 h of access during the same time of day (15). The same study also found that pullets with 5 h of access broken into two periods displayed compensatory water usage in the afternoon during their second water access period (15). Compensatory behavior could aim to fulfill behavioral and/or physiological needs, such that water restriction may contribute to dehydration or discomfort and reduce welfare (27). Our observations contradict findings by Hocking (14), where water-restricted birds did not display signs of thirst after a period of water deprivation despite limited or *ad libitum* access to feed. Decades of genetic selection since the previous work was published may have resulted in the behavioral differences observed in the current study.

### 4.2 Average bird water use

We hypothesized that SAD-fed birds would have higher water usage on feed days compared to ED-fed birds, specifically that SAD + ADLIB birds would have the highest water usage of all four treatments; however, this was not the case. During the on-feed day, SAD-fed birds in both ADLIB and WR groups used more water at 10 weeks but not at any other age. In fact, ED + ADLIB birds used more water than SAD + ADLIB birds at both 16 and 22 weeks on the on-feed day. These results differ from previous research that observed that SAD-fed pullets displayed more drinking behavior than ED-fed pullets at 11, 13, and 17 weeks (8). All pullets in the current study were switched to developer diets 16 weeks, so the increase in water use by ED-fed pullets could indicate a greater metabolic demand for water in this dietary phase. Indeed, Nielsen et al. (13) noted that higher levels of insoluble fiber (ISF) in an ED feeding regimen reduced water consumption in breeders. We observed a similar pattern between dietary fiber levels and average bird water intake for the ED-fed pullets, where higher fiber in the grower diet compared to the developer diet resulted in lower water use at 10 weeks during the grower diet phase and higher water use at 16 weeks during the developer diet phase. However, it is still not clear why the ED-fed pullets used more water than SAD-fed pullets as the ED diet contained higher ISF compared to the SAD diet in both diet phases. Inconsistencies between the results of the current study compared to Nielsen et al. (13) could be attributed to different genetic strains being used, as genetics is one of many factors that affects water use in chickens (28). Ogunji et al. (29) found that, when comparing two common broiler breeder strains, one displayed more water intake and fecal moisture despite feed treatment, suggesting that genetics may exacerbate water use patterns than feeding regimens alone.

Taken together with the behavior results described above, ED pullets spent less time at drinkers than SAD pullets later in the on-feed day, despite using more water overall. Consequently, ED pullets’ higher water use is driven by more drinking behavior during HAF than SAD pullets. The smaller feed portions may leave ED-fed pullets initially less sated, and higher water use shortly after feeding may be a strategy to increase gut fill. Correspondingly, ED-fed pullets displayed a higher W: F than SAD-fed pullets at 4, 16, and 22 weeks, demonstrating that ED pullets are using more water than indicated by their feed quantities compared to SAD-fed pullets. This could be a result of the inclusion of ISF in the ED diets, which has a higher water-holding capacity and allows for greater gastrointestinal distention with the presence of water to improve satiety with small feed quantities (30). Alternatively, higher water use may also be an indicator of frustration. Increased drinker use in feed-restricted birds is often regarded as a redirected stereotypic behavior associated with unsatisfied consummatory behavior (4). Although other behaviors were not measured in the present study, ED-fed birds displayed greater activity levels prior to feeding and lower performance of comfort behaviors compared to SAD-fed birds in previous research, which further supports potential distress for birds on this feeding program (8, 11).

Water restriction protocols resulted in no differences for water use between SAD and ED pens at 16 and 22 weeks. Implementing a water restriction protocol leveled out the amount of water use across both feed treatments. This could be concerning from an animal welfare standpoint if pullets cannot consume the amount of water they need, particularly for ED birds at 16 weeks where ED + ADLIB used more water than ED + WR. Bennett and Leeson (16) also noted the limiting effect on water use from water restriction protocols.

At 10 weeks, both SAD treatments used more water than both ED treatments during the on-feed day, and the SAD + ADLIB treatment used a similar amount of water as the ED + ADLIB treatment during the off-feed day at this age. The reason for this age-related change in water use at 10 weeks is not entirely clear. Previous work has attributed higher drinking behavior SAD-fed pullets to acclimating to their feeding program for a longer period of time than ED-fed pullets (8). At 10 weeks, pullets had been on their feeding program for 7 weeks, and no differences in water use were observed at 4 weeks of age after changing to the grower diet. As a result, the role of acclimation does not seem likely. It is important to note that though SAD treatments had increased water use at 10 weeks, their W: F was similar to ED treatments at this age and may indicate that increased water use is proportional to increased quantities of feed received by SAD pullets in comparison to ED pullets. However, further research is still needed to investigate why certain ages have increased sensitivity to feeding programs. There are few prior studies that examined drinking behavior and water use across an entire rearing period. Age specific strategies for breeder feed restriction and water management should be explored. Repeatability of this experiment should also be investigated in an environmentally controlled house.

### 4.3 Litter moisture

We hypothesized that the SAD feed treatments would have higher litter moisture, particularly under the water line, as a result of water wastage from increased drinker use. We also expected WR treatments to have lower litter moisture levels; however, this was not entirely the case. ED + ADLIB and ED + WR pens had greater WL litter moisture than SAD + ADLIB and SAD+WR pens at 16 weeks, while ED + ADLIB simultaneously used more water than SAD + ADLIB pullets at this age. Similar results were reported in Arrazola et al. (31) showing that birds fed a diet with increased ISF, similar to the ED diet in the current study, had higher litter moisture and worse footpad condition in earlier rearing phases. This previous study did not measure water intake or fecal moisture, so the cause for higher litter moisture and subsequent worsening of footpad condition is not entirely clear. The litter moisture results in the current study, combined with average bird water use, suggest that the ED diet may have created a greater physiological need for water than the SAD diet at older ages, particularly as they shifted onto the developer diet phase at 16 weeks. In addition, results from the current study suggest that water restriction protocols were not effective in mitigating litter moisture under the water line. These results differ from van Emous (15), which reported lower litter moisture under the water line with more restricted water access (3 h versus 5 h and 8 h of access). The previous study’s drinker systems contained drip cups under the nipple drinkers, whereas the drinker systems used in our study did not. The drip cups likely mitigated effects on litter moisture in van Emous (15).

However, SAD + ADLIB and SAD+WR pens had higher WL litter moisture at 22 weeks than both ED treatments, despite ED + ADLIB pullets using more water at this age than SAD+ADLIB pullets. Again, water restriction protocols were not effective in mitigating litter moisture under water lines, suggesting these protocols have limited utility for managing litter moisture. SAD+ADLIB pullets also displayed longer durations of drinking behavior at 12:00 and 14:30 at this age, so higher WL litter moisture content could be attributed to water spillage from drinker pecking. The ambient temperature increased at 22 weeks as the study entered into warmer summer months. Alongside greater portions of feed given to SAD birds during on-feed days, longer drinking durations and higher WL moisture at this age could be related to an increased thermoregulatory need to cool off using water as higher temperatures and higher feed intake are known to cause increased water usage in chickens (25).

ADLIB birds had greater GEN litter moisture across all ages. While the WL litter moisture is more indicative of water spillage, the GEN litter moisture is more indicative of fecal moisture content since this sample was taken 1 m away from water lines. Because ADLIB treatments tended to have increased water intake, particularly on off-feed days, this could cause an increase in fecal moisture which would result in a greater GEN litter moisture. SAD birds also had higher GEN litter moisture at 10 weeks, which is congruent with an increase in pullet water use at this age, again possibly causing higher moisture in both feces and litter. Ogunji et al. (29) found a similar association between increased water intake and higher fecal moisture. It is worth noting that nearly all GEN estimates in the present study do not exceed the optimal litter moisture of 25%, and none of the GEN estimates reach the concerning moisture range of 35–45% for caking (32). Despite treatment differences for GEN litter moisture, the estimates detected do not indicate animal welfare or environmental concerns.

### 4.4 Footpad condition scoring

We anticipated that treatments with the most water use and highest litter moisture would have worsened footpad condition scores; however, this was not the case. There were no significant differences in footpad scoring across treatments or ages. These findings are likely due to the GEN litter moisture mostly maintaining optimal litter moisture throughout the study and not impairing footpads as a result. In contrast, previous work concluded that quantitative feed restriction caused polydipsia that consequently increased litter moisture and incidence of footpad dermatitis (6). There are mixed results on the effect of qualitative feed restriction on footpad condition in earlier studies, with some observing worsened footpad condition in ED treatments fed a higher concentration of ISF and others observing improved footpad condition in groups fed higher concentrations of ISF (31, 33). The non-significant results in the current study could also be attributed to differences in sampling methods, whereas Kittelsen et al. (20) sampled footpad scores post-mortem using a five-level scoring system and Arrazola et al. (31) scored footpad condition biweekly using a binary scoring system. The current study scored footpads two times throughout the study using a three-level scoring system. Using different scoring systems, the number of collection timepoints, and scoring footpad condition on live birds could potentially cause inconsistent results.

### 4.5 Body weight and uniformity

We hypothesized that birds under ED feeding and ADLIB water conditions would have higher body weights and lower uniformity. Broadly, there was no clear effect of water treatment on body weight. There was an inconsistent effect of feed treatment, however. ED treatments did have higher body weights than SAD treatments at several ages. This is similar to findings by Sweeney et al. (9) that also observed higher body weights in ED-fed birds compared to SAD-fed birds. In the current study, drinking behavior and/or water use were measured at 4, 10, 16, and 22 weeks. Although body weight is a factor that can affect water use in poultry, with larger birds typically consuming more water than smaller birds (34), a significant difference in body weights was only noted during the 22 weeks data collection. At this age, SAD treatments had higher body weights than ED treatments but lower water use. This, combined with the lack of significant difference in body weights between treatments at the other data collection weeks, indicate that higher body weights were not a driver of increased water use in the current study.

Lastly, there was broadly no effect of feed treatment and water treatment on uniformity, as CV only differed between treatments for 2 weeks out of the entire trial. Carneiro et al. (35) also noted no differences in feed restriction programs on CV. In contrast, Zuidhof et al. (36) found that ED diets decreased uniformity compared to SAD diets, while Sweeney et al. (9) reported that ED diets increased uniformity compared to SAD diets. Differences in uniformity results could be attributed to genetics, where Zuidhof et al. (36) and Sweeney et al. (9) utilized Ross 308 and Ross 708 strains, respectively, while Carneiro et al. (35) and the current study used Cobb 500.

A limitation of this study was that it was conducted in a curtain-sided house, and future pullet research should investigate water use in an environmentally controlled, solid-walled house to mitigate the influence of higher ambient temperatures. Additionally, treatments could not be randomly distributed throughout the house due to animal welfare and infrastructure considerations described in the methods. Possible quadrant differences in light intensity and/or temperature may have affected treatments differently, but these variables were not recorded for each quadrant. Future work should also include metrics of satiety and hydration to disentangle these factors from stereotypic behavior to better understand the underlying mechanisms of water use and drinking behavior.

## 5 Conclusion

Feed and water restriction protocols have a complex effect on the drinking behavior, water use, and overall welfare of broiler breeder pullets during rearing. Our study found that SAD-fed birds displayed more drinking behavior later in the day compared to ED-fed birds, who displayed more drinking behavior at HAF. The type of feeding program influenced the timing of pullet drinking behavior, which is important for considering the timing of water restriction applied for different feeding programs. Furthermore, ED-fed birds displayed more water usage later in rearing compared to SAD-fed birds when water was provided ADLIB. These findings indicate possible differences in physiological and/or behavioral needs between feeding programs that are challenging to disentangle in the current study, including satiety and hydration as influenced by feed quantities and ingredients, stereotypic pecking behavior, and ambient temperatures. The WR treatments performed increased compensatory drinking behavior when water access resumed and anticipatory drinking behavior before water access was turned off. Water restriction may inhibit birds from consuming water when physiologically needed, subsequently causing thirst and discomfort and reduced welfare. Litter moisture under the water line is at the greatest risk at older ages, potentially caused by water spillage from more drinking associated with higher ambient temperatures and larger feed quantities in SAD treatments in particular. Although the water restriction protocol leveled out water use at older ages, it did not mitigate effects on litter moisture under the water line, and most general litter moisture values across treatments were still within an acceptable litter moisture range. As a result, the rationale for using water restriction protocols to maintain ideal litter condition is not supported by our results.

## Data Availability

The raw data supporting the conclusions of this article will be made available by the authors, without undue reservation.

## Footnotes

^1^www.accuweather.com

^2^http://www.boris.unito.it/
