# Supplementary material for: The effect of feed and water provision strategies on broiler breeder pullet performance and welfare
Source: Front Vet Sci. 2025 Aug 8;12:1611967. doi: 10.3389/fvets.2025.1611967 (PMC12371928; doi:10.3389/fvets.2025.1611967)
Supplement: Supplementary file 1 [file Image_1.pdf]

Supplementary Material

1 Supplementary Figures

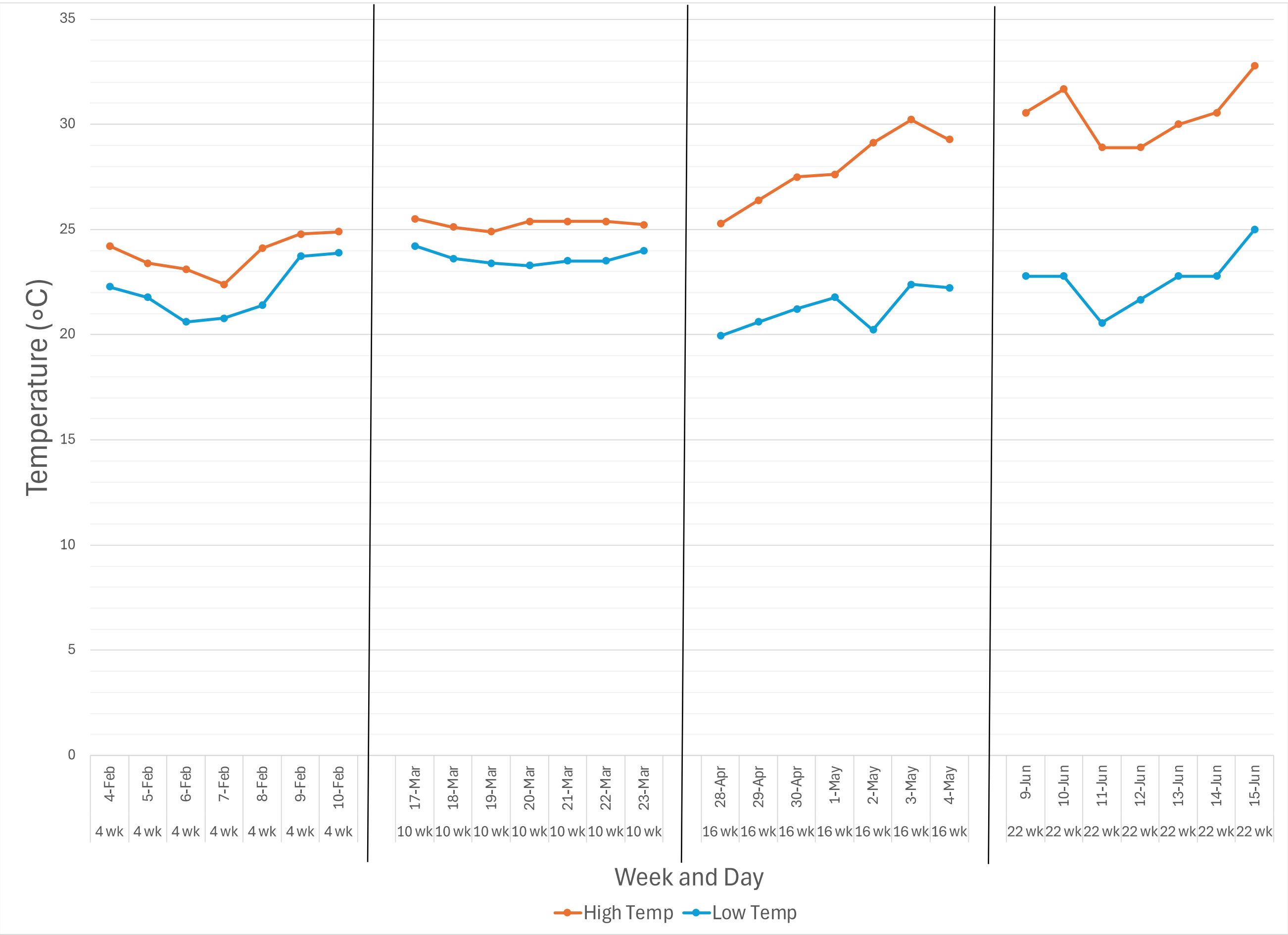

Supplementary Figure 1 High and low internal house temperatures (°C) during 4, 10, 16, and 22 wks. The thermometer for internal house temperatures was located on the every-day (ED) treatment side.
